# FBG Sensor for Contact Level Monitoring and Prediction of Perforation in Cardiac Ablation

**DOI:** 10.3390/s120101002

**Published:** 2012-01-17

**Authors:** Siu Chun Michael Ho, Mehdi Razavi, Alireza Nazeri, Gangbing Song

**Affiliations:** 1 Department of Mechanical Engineering, University of Houston, Houston, TX 77004, USA; E-Mail: gsong@uh.edu; 2 Division of Cardiology, Department of Medicine, Texas Heart Institute, Houston, TX 77004, USA; E-Mails: mehdirazavi1@gmail.com (M.R.); nazeri70@yahoo.com (A.N.)

**Keywords:** cardiac ablation, radiofrequency ablation, ablation catheter, transmural perforation, contact monitoring

## Abstract

Atrial fibrillation (AF) is the most common type of arrhythmia, and is characterized by a disordered contractile activity of the atria (top chambers of the heart). A popular treatment for AF is radiofrequency (RF) ablation. In about 2.4% of cardiac RF ablation procedures, the catheter is accidently pushed through the heart wall due to the application of excessive force. Despite the various capabilities of currently available technology, there has yet to be any data establishing how cardiac perforation can be reliably predicted. Thus, two new FBG based sensor prototypes were developed to monitor contact levels and predict perforation. Two live sheep were utilized during the study. It was observed during operation that peaks appeared in rhythm with the heart rate whenever firm contact was made between the sensor and the endocardial wall. The magnitude of these peaks varied with pressure applied by the operator. Lastly, transmural perforation of the left atrial wall was characterized by a visible loading phase and a rapid signal drop-off correlating to perforation. A possible pre-perforation signal was observed for the epoxy-based sensor in the form of a slight signal reversal (12–26% of loading phase magnitude) prior to perforation (occurring over 8 s).

## 1. Introduction

### 1.1. Background

The heart is incredibly durable, having to reliably sustain about 36 million pumping cycles during the lifespan of a normal healthy person [1]. However, various forms of heart disease compromise the functions of the heart and thus the longevity of the patient. One of the most common forms of heart disease is cardiac arrhythmia, in which the normal rhythm of the heart is disrupted by a period of disorganized contractions.

Atrial fibrillation (AF) is the most common type of arrhythmia, and is characterized by the disordered contractile activity of the atria (top chambers of the heart). In the United States, one fourth of adults over 40 years old will suffer from AF. Persons affected by AF are at a heightened risk of death, heart failure, and cardiac-related stroke [2].

A popular treatment for AF and cardiac arrhythmia in general is radiofrequency (RF) ablation. During RF ablation for AF, a catheter is passed venously into the heart, and is delivered into the left atrium through trans-septal puncture. Once contact has been made with defective tissue that caused AF, an electrode system integrated at the catheter tip transmits RF energy into the targeted tissue. Targeted cells warm up due to resistive heating and die as the temperature increases past 50 °C [3].

However, despite the usual success of RF ablation, several complications may occur, and some are life-threatening if not treated quickly. In about 2.4% of cardiac RF ablation procedures [4], the catheter is accidently pushed through the heart wall due to the application of excessive force. The resulting perforation in the heart wall allows the leakage of blood from inside the heart to the pericardial sac that surrounds the heart. Cardiac tamponade occurs when a sufficient amount of fluid accumulates in the pericardial sac to impair the functions of the heart. Usually, only a few minutes elapse between the start of perforation and when the life-threatening symptoms of cardiac tamponade manifest. Methods that allow detection of perforation or even a warning of impending perforation will be a valuable asset for cardiac RF ablation.

### 1.2. Literature

One of the most common techniques used to monitor cardiac RF ablation is fluoroscopy. Fluoroscopy is readily accessible and provides a visualization of catheter positions from many angles around the patient. However, fluoroscopy provides little information about the level of catheter-endocardial contact and at the same time increases the malignancy risk of both patient and physician [5–8]. Non-fluoroscopic techniques have been developed in the recent years that help to characterize the level of catheter-endocardial contact during RF ablation surgery.

Intracardiac echocardiography (ICE) was introduced to the RF ablation procedure to reduce the limitations of fluoroscopy. In ICE, an ultrasonic transducer is delivered through a 10F catheter (8F catheters are being made available) [5] to the various heart chambers. New developments (e.g., miniaturization) of this technology have led to transesophageal echocardiography (TEE), where the probe is placed into esophagus to allow enhanced imaging of certain parts of the heart [9,10]. The positioning of the ablation catheter within the heart can be projected onto a two dimensional plane, although fluoroscopy may be used in parallel to ensure the safe maneuver of the ablation catheter.

Non-imaging methods have also been developed to determine catheter-endocardial contact. Cao *et al.* investigated the use of electrical impedance between the tip electrode and the dispersive electrode to verify the contact state of the catheter [11,12]. The electrical resistive difference between blood and endocardial tissue served as a basis that allowed the operator to gauge the insertion depth of the catheter and qualify how firmly the catheter was pressed against the heart wall [13]. Demos and Sharareh meanwhile assessed various parameters of the RF ablation operation via optical spectroscopy. Near-infrared (NIR) light was transmitted through a fiber at the tip of the catheter, and another fiber received the reflected light. The difference in the spectral characteristics of the transmitted and received light provided information such as contact level, lesion quality, char formation, *etc*. [14].

Recently, a new type of ablation catheter (Tacticath) was developed to sense the contact force on the catheter tip. Three fiber Bragg gratings (FBGs) were integrated with a flexible unibody located near the catheter tip. As the unibody deformed, strains were transferred to the FBGs, which provided the signals needed to calculate the force vector [15,16]. Later studies determined the force needed to perforate each chamber of an explanted porcine heart [17].

All of the above sensors and techniques enhanced the ability of the operator to ascertain the progress and quality of a cardiac RF ablation procedure. However, despite the ability to measure various parameters of the procedure (e.g., contact force, lesion formation) there has yet to be any data establishing how cardiac perforation can be reliably predicted. As seen from the current literature, the capability to predict cardiac perforation is still quite undeveloped. The following sections describe the design of new FBG based sensor prototypes that (1) allows monitoring of the catheter-endocardial contact level, and (2) investigate signal characteristics that can predict or indicate transmural perforation of the left atrium in a living specimen.

## 2. Sensor Design

### 2.1. FBG Sensing Principle

FBGs are categorized as a type of in-fibre grating sensor. A small section of the optical fiber is inscribed with periodic perturbations (gratings) of refractive index. As broad band light is introduced into the fiber, a certain wavelength (*i.e.*, Bragg wavelength) corresponding to the period of the gratings is reflected back towards the source. The period of the gratings is affected by strain, temperature, and pressure. Any changes in those parameters are represented by a directly proportional shift in the wavelength of the reflected light, as indicated by Equation (1) [18]:

(1) $$\frac{\Delta \lambda_{B}}{\lambda_{B}}=(\alpha +\zeta )\Delta T+\left(1-P_{e}\right)\Delta \varepsilon$$

where *λ_B_* is the Bragg wavelength, *T* is the temperature, *ε* is the strain, *α* is the expansion coefficient, *ζ* is the thermo-optic coefficient, and *P_e_* is the photoelastic constant [18]. A pressure term that makes use of the fibers Poisson ratios may be added if pressure becomes an issue. For typical FBGs, the change in wavelength per strain is approximately 1.21 pm/με [19]. The temperature sensitivity of the FBGs used in this study was experimentally determined to be around 11 pm/°C. FBG sensors have been applied for use in a wide variety of novel uses in medicine, structural health monitoring, environmental monitoring, *etc*. [20–26].

### 2.2. Sensor Construction

**Figure 1.**
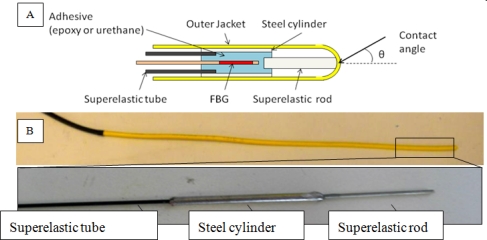
(**A**) Schematic of the sensor head; (**B**) Photos of the sensor exterior and interior.

An FBG (15 mm) was encased inside a short (3.5 cm) stainless steel cylinder (1.587 mm OD × 0.762 mm ID) (Figure 1). Two types of adhesives, epoxy (Devcon 5 min) and urethane (U1 urethane) were used separately to bond the FBG to the cylinder to test their respective strain transfer behavior. A superelastic tube (0.71 mm OD × 0.51 mm ID) protected the rest of the fiber, while a thicker superelastic rod (0.6 mm diameter, 10 mm length) was bonded to the distal tip of the cylinder to act as a strain transfer member. The superelastic tube was inserted into the steel cylinder approximately 3 mm while the rod was inserted about 2 mm into the cylinder on the opposite side. The FBG was fully encased inside the cylinder, with the distal tip of the fiber less than 1 mm away from the superelastic rod. All components were then housed into an OFNR fiber optic jacket (2.6 mm OD × 1.8 mm ID). As an important step to prevent blood infiltration to the components, the jacket was melted over the superelastic rod to create a blunt tip. Overall, the size and flexibility of the sensor were similar to an 8Fr ablation catheter.

Pressing of the catheter tip against a surface will produce a force vector that acts upon the superelastic rod. The force vector comprises of axial and lateral components, which correspondingly translate into compressive and tensile strains experienced by the FBG. Compressive and tensile strains correspond to negative and positive shifts in the Bragg wavelength, respectively. However, at this stage, a separation of the two forces has not yet been implemented; in other words, the acquired data (see Results section) is the superposition of these strains. Thus, the more perpendicular the catheter is to the surface, the more the Bragg wavelength will tend to shift downwards due to the dominance of compressive forces. Buckling and high contact angles (*θ* in Figure 1(A)) will induce lateral force components as well as encourage bending at the tube-cylinder junction and also at the cylinder itself, thus producing positive wavelength shifts during contact.

## 3. Experimental Setup

**Figure 2.**
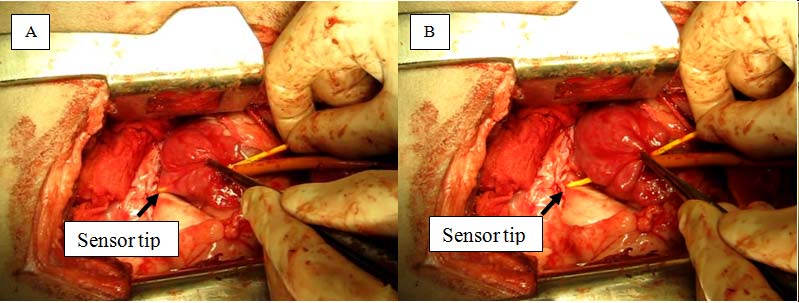
(**A**) Pushing of the sensor through the left atrial wall. The sensor tip is visible through thin tissue; (**B**) Completion of perforation. Forceps were used to assist this particular perforation.

Two live male sheep were used during the study. A thoracotomy was performed to gain direct access to the heart (Figure 2). The pericardial sac was partially excised to reveal the epicardium of the left atrium. Small incisions were made on the left atrium to allow entry for sensors, and purse strings were tightened around the wound to reduce blood loss. For the initial part of the experiment, the catheter alternated between resting in the space of the chamber or perpendicularly pressing against the endocardial surface. Towards the end of the surgery, the catheters were pushed through the wall of the left atrium to induce mechanical perforation of the left atrial myocardium. Actively used sensors were grasped directly about six to nine cm from the tip, depending of the insertion depth of the sensor. Occasionally, forceps were used to grasp the left atrial epicardium and pulled back onto the sensor to facilitate perforation.

Data acquisition was accomplished through a PC laptop connected to an optical interrogator (sm420 and sm130). A sampling frequency of 100 Hz (±2 Hz for sm420) was used during the operation. Dictation from the surgeon allowed correlation of observed FBG signals with events occurring on the operating table.

## 4. Results

### 4.1. Contact

**Figure 3.**
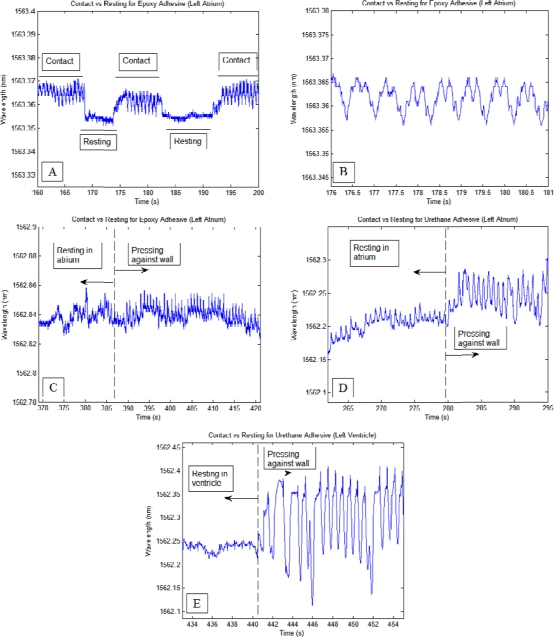
(**A**) Clear patterns in rhythm with heart beats were seen during firm contact of the epoxy-based sensor with the endocardial surface; (**B**) A close up of (A) showing clear and defined heart-beat patterns during firm contact with the heart wall; (**C**) Although the signal was not completely flat prior to contact, the presence of the heart-beat induced pattern signified that contact was made; (**D**) Similar behavior was observed for the urethane-based sensor, however, due to its high sensitivity, movements at the entry point produced weak period signals; (**E**) Contact monitoring using the urethane-based sensor while in the left ventricle. With stronger tissue, stronger contact can be made, which resulted in higher magnitude signals.

Each time the sensors were pressed firmly with the endocardial surface, clear patterns corresponding to myocardial contractions were observed. Once the sensor was retracted back into the chamber space, the patterns were diminished completely for the epoxy-based sensor and significantly diminished for the urethane-based sensor (Figure 3).

Generally, it was observed for the epoxy-based sensor that regular sinusoid signal patterns (contraction frequency slightly higher than 1 Hz) with and amplitude greater than or equal to 0.01 nm was sufficient to indicate firm contact with the heart wall. For the urethane sensor, the threshold was greater due to higher sensitivity, requiring amplitudes greater than 0.05 nm to ensure reasonable confidence that firm contact has been made. The higher sensitivity of the urethane-based sensor allowed whole-body vibrations and operator handling of the sensor to be mixed with the signal; and often whole-body vibrations were in synchronization with contraction rhythms, thus increasing the contact detection threshold. Thicker tissue allowed higher contact forces as seen in (Figure 3(E)) since thicker tissue can withstand higher pressures. Each contact event during the study involved firm pressure between the catheter tip and the endocardial surface, and was described often-times by the operator as being “near perforation”.

### 4.2. Perforation

Transmural perforation of the left atrium using the catheters was generally characterized by a distinct loading phase (greater than 0.07 nm change for the epoxy-based sensors and between 0.06 nm and 0.15 nm for the urethane-based sensor) and a rapid return to prior wavelength levels. The type of adhesive utilized for the FBG-steel cylinder bond led to different characteristics of the perforation signal profile. Myocardial contractions were more apparent during the loading process when the epoxy-based sensor was used.

**Figure 4.**
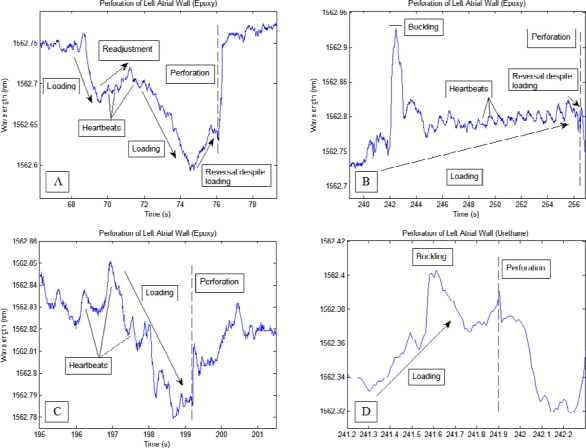

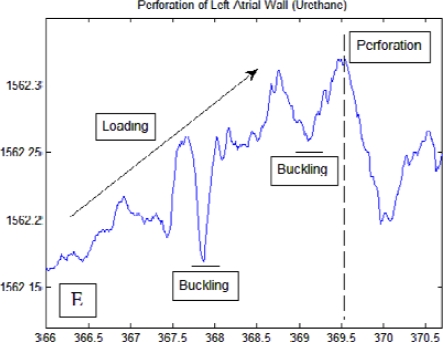
Transmural perforation of the left atrial wall using the fabricated sensors. (**A**) Epoxy-based sensor; note the negative loading phase and the slight reversal in signal immediately before perforation (**B**) Epoxy-based sensor; note the positive loading phase and a reversal prior to perforation. (**C**) Epoxy-based sensor; perforation occurred within 2.5 s of loading. The presence of a reversal may be mixed with heartbeat signals. (**D**) Urethane-based sensor; perforation marked by sudden drop in signal wavelength. (**E**) Urethane-based sensor; loading leading up to perforation followed by a more gradual drop off in signal wavelength.

During two perforation events using the epoxy-based sensor, a reversal of the loading phase (approximately 25% and 12% of loading phase Figure 4(A,B), respectively) occurred immediately before the onset of perforation. The loading phase spanned 8 s for Figure 4(A) and 16 s for Figure 4(B), while in Figure 4(C) the loading phase lasted about 2.5 s. This reversal was also observed during *in vitro* lab bench testing performed prior to this study. Urethane based sensors produced smoother perforation profiles in which heart beats were not readily seen compared to the rest of the signals. The signal drop-off during perforation was also more gradual compared to the epoxy-based sensor. Several peaks occurring during the loading phase of the urethane-based sensor correlated to the slight buckling of the catheter at the junction between the steel cylinder and the protective superelastic tube.

## 5. Discussion

### 5.1. Contact

As seen from the experimental data, the sensors were able to detect contact with the endocardial surface and were also able to provide a unique signal pattern corresponding to transmural perforation of the left atrium. The sensing ability of the sensor was greatly impacted by the type of adhesive used for bonding the FBG and the superelastic rod to the steel cylinder. Compared to urethane-based catheters, epoxy-based catheters were less sensitive to external input. This insensitivity filters out many disturbances, such as whole catheter vibrations, thus making the perforation event clearer than with urethane-based catheters. As seen in Figure 3(A), myocardial contractions appeared as rhythmic peaks in the signal whenever firm contact was maintained and were greatly diminished when contact was absent. These peaks can serve as a sufficient indicator that catheter-endocardial contact has been initiated. On the other hand, less information is available regarding the level of contact. Naturally, higher contact forces yield stronger signals, as seen when the catheter was passed into the left ventricle. However, while stronger tissue can withstand higher contact forces, weaker, thinner tissue such as those in the left atrium should not be subjected to similar levels of contact. Thus as a safety measure, the operator should be cautious not to push much further when the sensor signals present such peaks in the signal at or above the threshold levels (0.1 nm and 0.5 nm for epoxy-based and urethane-based catheters, respectively). Conversely, as these contact waveforms indicate firm catheter-endocardial contact, the operator should seek for their appearance before deciding on whether to proceed with ablation.

### 5.2. Perforation

Each perforation conducted during the study was characterized by a loading phase and a sudden, rapid return to prior wavelengths at the time of perforation. Negative loading phases correspond to axial loading of the catheter while positive loading phases correspond to the presence of lateral forces, or shearing at the catheter tip. Thus for the current sensor design, a positive wavelength change after reaching thermal equilibrium suggests that the catheter tip may not be fully perpendicular to the endocardial surface (e.g., Figure 4(B,D,E), during loading)). Of note in Figure 4(A,B) is the brief reversal of signal wavelength immediately prior to perforation despite continual pressure exerted by the operator. This reversal was also observed for the epoxy-based sensor during perforation bench tests using a slab of tissue simulant and a commercially available pig heart. An explanation may be that as the contact level started to reach perforation levels, the targeted tissue began to weaken and thin, temporarily relieving the pressure between the catheter tip and the atrial wall. The length of the reversal was more prolonged and apparent when the perforation was carried out more slowly; in fact, as seen in Figure 4(C) the reversal may be absent when perforation was carried out quickly (2.5 s in Figure 4(C) compared to greater than 8 s for Figure 4(A,B)). Short perforation times prevented correlation of perforation speed with the observability of a reversal signal. However, sensitivity-induced competition with secondary signals such as operator handling readily reduced the prominence and clarity of the perforation signal. Therefore, it may be concluded that stiffer adhesives such as epoxy will allow exactly the right strain transfer to isolate signals unique to perforation. On the other hand, one should realize that perforations performed in this study were carried out intentionally, and during actual ablation procedures, a loading phase may not be as apparent to the operator. For load-sensing enabled RF ablation catheters, Figure 4(B) may be more representative of more realistic situations in which the magnitude of the baseline and the heart beat induced peaks slowly magnified until a sudden wavelength drop-off occurred. Thus, during RF ablation, the operator should check for alternate signs of perforation whenever a wavelength drop-off is observed after steady increases in signal waveform magnitudes.

Perforation results from this study agree with the results presented by Shah *et al.* [17], with both studies showing a characteristic loading phase followed by a sharp drop in contact force coinciding with perforation (seen in the current study as a quick shift in Bragg wavelength to pre-loading levels). A contact force threshold (100 g) was recommended by Shah *et al.* to minimize the chances of perforation [17]. On the other hand, the brief reversal as seen in Figure 4(A,B) may act as an additional warning signal for operators of impending perforation.

The data acquired from this study lays the foundation for further improvements in sensor designs in subsequent studies. During the perforations shown in Figure 4(B,D,E), buckling of the sensor between the cylinder and the protective superelastic tube produced large peaks in the signal. Later designs will reinforce this junction to reduce the chance of buckling during loading. Furthermore, the thicknesses of the cylinders housing the FBGs were thin and thus susceptible to substantial bending stresses. Increasing the thickness will reduce the effect of bending stresses, but will impose obstacles in other aspects of the sensor, such as the size; thus a stiffer type of material may be used to lower this effect while maintaining a small size.

### 5.3. Effects of Temperature

As indicated by Equation (1), changes in temperature correlate to shifts in the Bragg wavelength but do not affect sensitivity to mechanical strain. Throughout the study, temperature changes occurred mainly from the movement of the sensor into and out of the cardiac chambers. Insertion of the sensor into the heart produced an upward shift of approximately 0.4 nm in the Bragg wavelength. Roughly 30 s were required for the sensor to reach thermal equilibrium once inside the heart (hence no more temperature-induced wavelength shifts). Extractions of the sensor lead to the recovery of the wavelength shift to original, pre-insertion levels.

Although temperature did not have an effect on the current results after the sensor reached thermal equilibrium within cardiac chambers, a compensating mechanism will be necessary if the sensor is to be calibrated for force. A stress-relieved FBG sensor placed close to the sensing area will be able to measure temperature-induced wavelength shifts while at the same time be isolated from contact strains (an example of this mechanism is shown in [27]).

## 6. Conclusions

Sensors fabricated in similar form as an ablation catheter were able to allow monitoring of catheter-endocardial contact levels and investigate any signal behavior that helps in predicting or indicating the occurrence of transmural perforation of the left atrium. Multiple, periodic peaks were seen in the signal when contact was made and quickly diminished in the absence of contact, thus establishing a condition indicating the presence of contact. Furthermore, perforation was identifiable by a baseline-shifting loading phase followed by a rapid return to prior wavelength levels. For epoxy-based sensors, a short signal reversal was observed prior to the moment of perforation, and it is recommended that stiff adhesives be used when investigating the characteristics of perforation. Knowledge of these signal characteristics will help future ablation procedures regarding the application of adequate pressure and also for reducing perforation incidences.
